# Preparing the body for psilocybin therapy: receptive and generative interoception in cancer patients

**DOI:** 10.3389/fpsyt.2026.1890966

**Published:** 2026-10-08

**Authors:** Anthony L. Back, Cynthia J. Price, Bonnie A. McGregor, Andrew Markell

**Affiliations:** 1 Department of Medicine, University of Washington, Seattle, WA, United States; 2 Fred Hutch Cancer Center, Seattle, WA, United States; 3 Department of Biobehavioral Nursing and Health Informatics, University of Washington, Seattle, WA, United States; 4 Orion Center for Integrative Medicine, Seattle, WA, United States; 5 Dawn Collective, Bozeman, MT, United States

**Keywords:** cancer, contemplative practice, interoception, predictive coding, psilocybin, psychological therapy, psychosomatic medicine, somatic preparation

## Abstract

Cancer patients arrive for psilocybin therapy with bodies altered by illness and treatment — bodies that they have learned to brace against, dissociate from, or scan for threat. Research on psilocybin-assisted therapy for cancer-related depression and anxiety consistently identifies the quality of the acute subjective experience — reflected in constructs such as acceptance, surrender, boundlessness, and emotional breakthrough — as a key predictor of therapeutic benefit. Yet most preparation protocols focus on cognitive intention-setting and basic mindfulness while neglecting to address the body directly. We propose that anxiety and depression in this setting may involve a somatic substrate that has not been adequately specified. We distinguish two capacities to ‘feel into the body’ relevant to psychedelic therapy: interoception as perception (the perceptual access to bottom-up bodily signal) and interoceptive engagement (the broader capacity that links bodily perception to posture, breath, structural intent, and affective stance). We describe a six-category phenomenological taxonomy of somatic experiences in psilocybin therapy (exteroceptive, deep somatic, visceral, vestibular, nociceptive, emergent). We also map receptive practices, designed to improve interoception as perception, and generative practices, designed to improve interoceptive engagement. The framework is grounded in current predictive-coding accounts of interoception, but does not depend on any single neuroscientific model. An ongoing Phase 2 trial of group retreat psilocybin therapy for metastatic cancer patients will provide an initial test of our hypothesis that interoceptive training may influence therapeutic outcomes in psilocybin therapy for patients with cancer.

## 1 Introduction: preparation for psilocybin therapy for cancer often overlooks the body

In our prior studies of patients with metastatic cancer who receive psilocybin in a group retreat setting (1, 2), we have observed that some participants do not “drop in” (a term facilitators use for settling into the unfolding subjective experience rather than monitoring it from a distance) to the experience easily. One participant told us that they were expecting to see visual lights and distanced themselves from allowing other feelings because they assumed that they needed to see visual phenomena as part of entering their experience. Another participant began with concern that the psilocybin “wouldn’t work” and found themselves repeatedly asking “is this working?” and assessing their own mental status. Both of these participants later rated their experience with Mystical Experience Questionnaire (MEQ-30) scores that were lower than “complete”.

Our observation is that this difficulty “dropping in” to a psilocybin experience is related to their capacity to feel into their bodies. Experienced psychedelic facilitators sometimes describe this as a participant who stays ‘in their head’ (3, 4), as they attempt to think their way into the experience (“I should be seeing lights”) or analyze their experience (“It’s not working”). During body scans that invite them to “tune in” to areas of their bodies, we observed that they appeared to have more difficulty than expected in sustaining attention to bodily sensation. Published advice for participants from experienced guides routinely includes advice to “surrender”, “trust” and “let go” (5, 6). For participants without prior somatic training, these can function as high-level abstractions — words that name embodied stances but offer no path into them — leaving the body itself unaddressed. Other researchers have described difficulties related to entering a psilocybin experience (7–9) but to our knowledge the specific difficulty we describe here of ‘dropping in’, which we use to describe a patient stance of accessing, attending to, and monitoring bodily experience at the onset of a psilocybin session, has not been named.

Most contemporary preparation protocols emphasize cognitive and relational preparation; comparatively less attention has been given to systematic methods that explicitly target bodily awareness as a trainable capacity (10–12). Current guidelines direct practitioners to build a therapeutic alliance, elicit intentions, negotiate safety around practices such as touch, and provide psychoeducation about “flight instructions” (the brief verbal guidance offered to participants before a dosing session) that include the aforementioned advice to trust and let go. But we are not aware of an existing protocol that systematically trains the capacity to feel into the body, to train interoceptive capacity. Preparation protocols often rely on the assumption that pharmacological and psychological processes together will make the experience therapeutically useful, without systematically targeting somatic capacities as an independent preparation domain. The empirical research now demonstrates that how patients engage with their psychedelic experiences, operationalized as acceptance, surrender, boundlessness, or emotional breakthrough, is a key predictor of therapeutic benefit (13–17). In this paper, we argue that even when psilocybin relaxes ‘top-down’ beliefs, the patient must be able to receive and organize ‘bottom-up’ sensation. In the empirical literature, the felt sense of bottom-up signals has not been adequately specified, nor has the capacity to manage bottom-up signals been treated as a training target. Building on existing research showing that ‘bottom-up’ capacity can be trained, we propose preparation activities that are grounded in neuroscience, supported by evidence-based practice, and are empirically testable. We frame this preparation as an innovative therapeutic approach to the interplay between physical and mental health in serious illness. Our central, testable hypothesis is that training two distinct capacities — interoception as perception and interoceptive engagement — before dosing improves how patients receive and organize what the pharmacology may make available, and thereby its therapeutic benefit; an ongoing Phase 2 trial (BACK007) is designed to evaluate it.

The basic argument is as follows: psilocybin may loosen a patient’s predictive models, but that patient still needs the bodily capacities to receive, tolerate, and organize bottom-up somatic sensations. The purpose of this Hypothesis and Theory article is to specify a conceptual framework and a testable hypothesis for these bodily capacities. To illustrate what we mean, we use clinical vignettes from our completed trials of group retreat psilocybin therapy (1, 2). We consider these vignettes to represent anecdotal observations that explain the origins of our framework and hypothesis, not empirical research that proves our hypothesis. The paper makes three contributions: a six-category phenomenological taxonomy of somatic experience under psilocybin (Section 5); a distinction between two trainable somatic capacities (Section 6), interoception as perception and interoceptive engagement; and finally a description of preparation practices that are operationalized as a manualized protocol (Sections 8–9). From these we derive our central hypothesis — that training both capacities before psilocybin dosing could improve how patients receive and organize the bottom-up input that psilocybin makes available — together with three pre-specified, falsifiable predictions (H1–H3) under evaluation in an ongoing Phase 2 trial (BACK007).

## 2 Cancer patients’ capacity to feel into the body has been altered

Cancer patients arrive for psilocybin therapy already estranged from their bodies. Research describing the phenomenology of cancer patients demonstrates that many carry a sense of feeling betrayed by the body, leaving the body, or experiencing the body as alien or as enemy (18–22). Several cancer-specific factors produce this estrangement: surgery alters body schema (23), chemotherapy produces neuropathies and visceral disruption (24), radiotherapy can damage nerves in a way that produces chronic somatic noise (25, 26). Furthermore, oncology clinics often approach the patient’s body as a medicalized object to be scanned, tested, and subjected to treatments.

We hypothesize that these cancer-related changes may alter the brain’s model for interoceptive prediction (27, 28). By analogy with findings in chronic pain and depression (29, 30), we propose that these changes may lead patients to attenuate, avoid, or defensively reinterpret bodily signals, and perhaps even to suppress or override bodily signals that would cause distress. This is not just psychological; what has occurred is a remodeling of the brain’s predictive architecture—which we explain in detail in Section 10. Cancer patients may thus make a phenomenon visible that is also present, less severely, in other populations—chronic pain, trauma histories, depression, and aging—in which interoceptive capacity is similarly degraded.

We do not assume, however, that cancer is the sole origin of reduced interoceptive capacity in this population. Other factors may independently alter interoceptive processing and may be present in patients arriving for psilocybin therapy. Alexithymia — difficulty identifying and describing one’s own feelings — is the most extensively studied correlate of impaired interoceptive awareness and has been proposed to reflect a general deficit of interoception (31). Neurodevelopmental and neurodivergent conditions, including autism and attention-deficit/hyperactivity disorder, are associated with atypical interoceptive profiles (32, 33). Pre-existing trauma, depression, chronic pain predating the cancer diagnosis, and medication effects can be associated with altered interoception (30, 34–36). For our framework, that interoceptive capacity is reduced does not change the central prediction: that interoceptive capacity is trainable. For the interpretation of our pilot data, however, these factors constitute potential confounders (see Sections 9 and 11).

Furthermore, the institutional stance of patienthood typically cues patients to dissociate, brace against insult, or self-medicate—a pattern of contraction from the challenges of living with cancer, the work required to undergo cancer therapy, and the existential fact of one’s mortality (21, 37). Escape from this situation can be framed as individual struggle requiring family or group ‘support’ with temporary relief through diversions or treatment ‘vacations’, which do not address the underlying somatic conditions (38). Mindfulness techniques based on increasing ‘awareness’ often increase self-consciousness of these dynamics without improving the body’s capacity to feel under stress (39–41); in some participants, they can amplify rather than reduce defensive responses (40, 42). The result is psilocybin experiences that can be constrained or incompletely realized due to habitual defensive responses that originate in the body.

## 3 Existing interoception research does not address what psychedelic therapy requires

The term interoception originates with Sherrington (43), who described it as sensations from inside the body, especially the viscera. A more recent definition (44) is the process by which the nervous system senses, interprets, and integrates signals originating from within the body. The concept now encompasses a broad terrain—from cardiac and respiratory signals, through visceral and musculoskeletal sensation, to affective feelings and metacognitive awareness of internal states.

Much of the existing empirical literature on interoception has been dominated by experiments asking healthy volunteers to detect their own heartbeat, which measures a person’s ability to detect and track their own heartbeats under controlled conditions (45). This heartbeat paradigm, while methodologically well developed, captures a narrow slice of the range of somatic experiences our study participants report (46). Existing self-report instruments such as the Multidimensional Assessment of Interoceptive Awareness (MAIA-2) capture a series of interoception-related constructs: noticing, not-distracting, self-regulation, body listening, trusting, and others (47, 48). These instruments have clear value and will play a role in our empirical testing below. However, they are not phenomenologically mapped to psychedelic experience: a high MAIA-2 score tells us little about which somatic experiences a participant is prepared to encounter under psilocybin. Alexithymia, the field’s principal construct for impaired interoceptive access (31), names a global deficit; it does not specify which capacities are degraded or which somatic experiences a participant is unprepared to meet, which our taxonomy is designed to provide.

## 4 A phenomenologically grounded taxonomy specifies what preparation should train

The existing interoception literature lacks a phenomenologically grounded, clinically actionable taxonomy for psychedelic experience. (We use the term phenomenological to mean grounded in the first-person accounts of participants and facilitators.) In this paper we offer a six-category taxonomy of somatic experience in psilocybin therapy, derived from physiological research and clinical observation. The taxonomy reaches more broadly than the existing interoception literature, so we have called it here a taxonomy of ‘feeling into the body’—which is also a practical term that can be used with participants. Having this taxonomy enabled us to design a preparation protocol aimed to train the specific levels of interoceptive capacity needed for a psilocybin experience. The paper closes with the empirical plan under which we are testing the protocol in an ongoing Phase 2 trial.

We recognize that our work builds on a tradition of somatic approaches to psychological treatment—Somatic Experiencing (49), Sensorimotor Psychotherapy (50), Mindful Awareness in Body-Oriented Therapy (41), and somatic approaches to contemplative practice (51, 52)—which have long recognized the clinical relevance of somatic awareness and have influenced psychedelic therapy practice. Our approach has been informed by these predecessors yet differs in several respects. We have grounded our work in a predictive-coding framework linked to empirical accounts of interoception and Mindful Awareness in Body-oriented Therapy (MABT), an evidence-based approach designed to develop interoceptive capacity. From MABT we have drawn on a foundational structure, practices of body literacy, the use of guided attention to internal sensations, and the manualized sequence of practices (41). Here we add a physiologically-based taxonomy of somatic experience, and an approach purpose-built for the specific preparation needs of psychedelic therapy. Finally, our approach is designed with hypotheses that can be tested empirically within the context of research on psilocybin therapy.

## 5 A taxonomy of ‘feeling into the body’ for psychedelic experience

### 5.1 Principles of the taxonomy

Our taxonomy distinguishes six categories of somatic experience (**Table 1**). The six categories are distinguished by receptor basis where the physiology supports it, or by felt quality. In clinical observation, categories may co-occur and blur; the taxonomy is a clinical scaffold meant to improve recognition of somatic experiences, not a claim that somatic experience partitions cleanly, and not a validated instrument.

**Table 1:** A Taxonomy for ‘feeling into the body’.

Category	Receptor basis	Felt quality	Commonly used descriptive terms
Exteroceptive	Meissner & Pacinian corpuscles, Merkel cells, Ruffini endings, thermoreceptors, cutaneous nociceptors	Localized to skin surface; touch, pressure, temperature, sharp pain	tingling, prickling, buzzing, warm, cool, flushed, goosebumps, crawling, electric, itchy, tender
Deep somatic	Muscle spindles, Golgi tendon organs, joint mechanoreceptors, fascial mechanoreceptors, muscle metaboreceptors	Pressure, heaviness, tension, fatigue, stretch; located in muscles, joints, deep tissue	heavy, dense, tight, clenched, braced, releasing, softening, melting, solid, trembling, unwinding
Visceral	Gut stretch and chemoreceptors, cardiac mechanoreceptors, pulmonary stretch receptors; vagal and spinal visceral afferents	Diffuse, poorly localized, affectively valenced; “something wrong” with emotional tone rather than precise location	churning, knotted, hollow, full, queasy, fluttering, dropping, rising, blooming, contracting
Vestibular	Semicircular canals (rotational), otolith organs (linear acceleration, gravity)	Groundedness, weight dropping, spatial orientation, feeling “held” by gravity; often below conscious awareness	spinning, tilting, floating, falling, grounded, anchored, rooted, suspended, drifting, weightless
Nociceptive	Cutaneous, deep, and visceral nociceptors; central pain processing; protective motor patterns	Pain and alarm across multiple somatic levels; protective-defensive response patterns	sharp, burning, piercing, throbbing, crushing, tearing; bracing, guarding, flinching, freezing
Emergent	No identified receptor class. Candidate mechanisms: autonomic co-activation, global neuromodulator shifts, fascial mechanotransduction, interoceptive inference dynamics	May be localized (e.g., heart center) or pervasive (e.g., oceanic). Somatic in felt quality but not traceable to a specific receptor class	aliveness, stillness, spaciousness, luminosity, dissolving, oceanic, resonance, porosity, streaming

We have identified six categories of ‘feeling into the body’ with representative descriptor vocabulary for each category. We deliberately did not call this a taxonomy of interoception because some categories fall outside current accepted definitions of interoception (44). We view the vocabulary descriptors as language that can be used to expand the range of ‘in the body’ experiences that participants and facilitators alike can identify, talk about, and make meaning from. This taxonomy is a clinical scaffold, not a validated instrument. We do not intend these terms to be used to ‘correct’ participants or enforce their ‘proper’ use.

### 5.2 Exteroceptive (cutaneous)

The exteroceptive category encompasses sensations at the skin surface—touch, pressure, temperature, sharp pain (53). The receptor basis is well understood: Meissner’s and Pacinian corpuscles, Merkel cells, Ruffini endings, thermoreceptors, and cutaneous nociceptors. Felt qualities include tingling, prickling, buzzing, warmth or coolness, flushing, goosebumps, and crawling sensations. Under psilocybin, exteroceptive experiences are less commonly foregrounded than the other categories, but they are familiar to most people and serve as a useful starting point for training: they are the ‘feeling’ modality closest to ordinary waking experience.

### 5.3 Deep somatic (musculoskeletal, fascial, proprioceptive)

The deep somatic category encompasses sensations arising from muscle, fascia, joints, and related structures (53, 54). The receptor basis includes muscle spindles, Golgi tendon organs, joint mechanoreceptors, fascial mechanoreceptors, and muscle metaboreceptors. Felt qualities include heaviness, density, tightness, clenching, bracing, releasing, softening, melting, trembling, and unwinding. Deep somatic experiences are among the most commonly reported under psilocybin—shaking, trembling, release of muscular tension, felt unwinding of long-held postural patterns—and for many participants they are relatively easy to localize.

### 5.4 Visceral (organ and cavity)

The visceral category encompasses sensations arising from internal organs and body cavities (55). The receptor basis includes gut stretch and chemoreceptors, cardiac mechanoreceptors, pulmonary stretch receptors, hepatic osmoreceptors, and vagal and spinal visceral afferents. Felt qualities include churning, knotted, hollow, full, queasy, fluttering, dropping, rising, clenching, blooming, opening, contracting, and sinking sensations. Visceral experiences are characteristically diffuse, poorly localized, and affectively valenced—often experienced as “something wrong” with an emotional tone rather than as a precisely locatable sensation. This is partly a consequence of the anatomical distribution of stretch receptors in the organs involved. Visceral sensations may be the most common somatic dimension of psilocybin experience, and gastrointestinal discomfort, nausea, and abdominal unease are among the most common somatic side effects.

### 5.5 Vestibular (gravitational and spatial)

The vestibular category encompasses sensations of spatial orientation, gravity, and bodily position in space (53, 56). The receptor basis is the vestibular apparatus: semicircular canals for rotational acceleration, otolith organs for linear acceleration and gravity. Felt qualities include spinning, tilting, floating, falling, grounded, anchored, rooted, suspended, drifting, and weightless sensations. Vestibular experiences often operate below conscious awareness in ordinary life but become prominent under psilocybin, where they underlie out-of-body experiences, feelings of floating or flying, and, conversely, experiences of being profoundly grounded or “held” by gravity. The vestibular system provides a spatial reference for bodily awareness; its disruption is implicated in distorted body image and feelings of disembodiment (56).

### 5.6 Nociceptive (pain, alarm, protective)

The nociceptive category encompasses pain and alarm sensations across multiple receptor systems (53). Felt qualities include sharp, searing, burning, piercing, throbbing, splitting, crushing, tearing, and radiating sensations. Nociceptive experience also includes protective-defensive response patterns: bracing, guarding, flinching, armoring, retreating, shutting down, going blank, freezing. Pain can be felt across multiple somatic levels and is not confined to a single receptor class. While overtly painful experiences are uncommon during well-held psilocybin sessions, we include nociception in the taxonomy because of the prevalence of cancer pain in our study population and because nociceptive-protective patterns frequently co-occur with other categories of somatic experience under psilocybin.

### 5.7 Emergent (gestalt, field, energetic, whole-body)

The emergent category encompasses whole-body-state experiences that cannot be localized to physiologically defined sensations or individual sensory channels (57). No identified receptor class underlies these experiences. Possible explanatory frameworks include autonomic co-activation patterns, global neuromodulator shifts, and the dynamics of interoceptive prediction itself. Felt qualities include aliveness, stillness, spaciousness, presence, absence, coherence, fragmentation, luminosity, being-breathed, dissolving, crystallizing, oceanic, resonance, porosity, streaming, flowing. The category includes experiences common in psilocybin sessions: profound boundlessness, spatial expansion (such as a heart “exploding out of the chest”), merging with surroundings, and felt continuity with natural elements such as trees, water, or mountains.

We include this category because these experiences are widely described in the psychedelic phenomenological literature (5, 58) and occur frequently in our clinical observations, so we consider them to be clinically salient in psychedelic therapy even though their receptor basis is undetermined. We take an agnostic stance on ontology and a committed stance on clinical utility: regardless of what emergent experiences ultimately are, they occur, they matter therapeutically, and facilitators need vocabulary and concepts to work with them. Participants from studies of MABT commonly report emergent qualities in their experiences of sustained interoceptive awareness (59). Comparable phenomenological descriptions appear in contemplative traditions in which emergent experiences are described using tradition-specific terms—chi in Chinese medicine (60), prana in yogic tradition (61), or anatomically-located concepts such as dan tien (62)— that function as emergent-category descriptors within those frameworks (63). These descriptors are each embedded in their own cosmology and framework, and we are not suggesting they are interchangeable with our taxonomy or with each other. We invoke them here only to note that whole-body, non-localizable somatic experience has been described, though interpreted differently, across diverse contemplative lineages.

### 5.8 What the taxonomy is and isn’t

Most interoception literature, with its focus on heartbeat detection and breath awareness, does not address all the somatic layers (deep tissue, visceral, vestibular, emergent) that participants report from psilocybin experiences. This taxonomy is clinically actionable, hypothesis-generating, and organized around categories that can guide both preparation exercises and in-session clinical observation. It is not psychometrically validated, not exhaustive, and not a claim that somatic experience is naturally carved into exactly six kinds. We expect the taxonomy to be refined as empirical work progresses—some categories may split, others merge, and the descriptor vocabulary will develop through participant use. We offer it as a starting framework that is specific enough to do clinical work and provisional enough to be corrected by data.

## 6 Two modes: ‘interoception as perception’ and ‘interoceptive engagement’

The taxonomy described in the previous section maps the structure of ‘interoception as perception’, by which we mean the various types of bottom-up signals that constitute the raw material of bodily awareness. What the taxonomy does not address is how the participant relates to that signal, which we call their ‘interoceptive engagement’.

### 6.1 Why the distinction between the two modes matters

For example, one of our study participants felt in her psilocybin session a profound relaxation of her entire musculature, which for her translated into a deep sense of safety that she embraced, and revisited during her daily meditations, with a resulting profound influence on her daily well-being. A different participant felt in his psilocybin session that he could fly around the room, and had a bird's-eye view of himself, his life, and his cancer. Yet he treated his feeling of lightness and nimbleness as a one-time, drug-induced passing curiosity, so the change in his perspective that he visited temporarily did not persist. These examples suggest that the properties of the bottom-up signals from the body alone are not sufficient for lasting change. Both patients perceived interoceptive signals, but only one of the patients engaged with those interoceptive signals. Our observations suggest that the patient’s interoceptive engagement, their stance toward bottom-up signals from the body, is an independent variable that influences change.

The distinction between ‘interoception as perception’ and ‘interoceptive engagement’ is not captured in the dominant model that explains how psilocybin appears to produce its therapeutic effects. The dominant REBUS model (64) proposes that psilocybin relaxes the precision-weighting of high-level priors, permitting ascending bodily and affective signal that is normally suppressed by top-down predictions to enter awareness with unusual force. The therapeutic potential of the experience derives from this enhanced bottom-up access — material previously held outside awareness becomes available for processing and integration. The REBUS model does not specify what happens to bottom-up signals. (We develop these mechanisms more fully ahead in Section 10.)

But our clinical examples also suggest that not all bottom-up signal is therapeutically equivalent. Whether bottom-up signals produce clinical benefit depends on at least three properties of the signal-and-system interaction: the precision of the signal itself, the magnitude of the prediction error it generates against existing priors, and the affective valence with which it is registered. Signal that is too intense, too discrepant from the system’s existing model, or too strongly tagged as threatening may be difficult for the participant to integrate into adaptive psychological change, and may be experienced as overwhelm and can lead to the phenomenology of difficult or harmful experiences.

The clinical task of preparation, then, is not only to expand the participant’s perceptual access to bottom-up signal but to develop the somatic and attentional conditions under which intense signal can be received as information rather than threat. This second task — preparing the body to metabolize what the pharmacology will make available — is what the engagement construct names and what generative practice trains.

Thus we propose distinguishing between *interoception as perception* — the established construct in the contemporary literature, referring to the perceptual access to ascending bodily signal — and *interoceptive engagement*, the broader capacity that couples interoceptive perception to posture, breath, structural intent, and affective stance.

### 6.2 Interoceptive engagement has two modes

Within interoceptive engagement, *expansion* and *contraction* name two clinically central modes, though probably not the only ones. Expansion describes a state in which the body remains structurally available under the pressure of intense bodily signal: the spine lengthens rather than collapses, the diaphragm continues to move freely, peripheral awareness remains intact alongside central attention, behavioral repertoire stays accessible. Contraction describes the opposite: trunk and jaw tense, breath shortens and rises, attention narrows, postural openness collapses, the available repertoire shrinks toward bracing, fleeing, or shutting down. These patterns may be understood both as autonomic configurations and, phenomenologically, as habitual bodily stances — habitual ways the participant meets bodily signal, learnable and modifiable through practice.

We acknowledge that ‘interoceptive engagement’ is related to an existing construct, ‘interoceptive experience’. Price & Weng use ‘interoceptive experience’ to describe what a participant feels when interoceptive attention is sustained, which we characterize as sustained detection of bottom-up signals from the body (59). We are using ‘interoceptive engagement’ to describe the stance a participant takes toward bottom-up afferent signals (from posture, or breathing, or movement, for example): does the participant open and even increase their weighting of bottom-up signals (expansion, as described in the previous paragraph)? Or does the participant contract and reduce their weighting of bottom-up signals (contraction)? We consider interoceptive engagement to be operationalized through the weighting of bottom-up signals, so that this term includes what the body does weighting bottom-up signals *and* detection of bottom-up signals. We propose that interoception training for psilocybin preparation needs to address the participant’s capacity for interoception as perception, and also their capacity for interoceptive engagement.

### 6.3 How interoception maps onto previously identified outcome predictors

Our distinction between interoception as perception and interoceptive engagement has empirical traction in factors identified in prior psychedelic studies, though it has not previously been articulated in interoceptive terms. Acceptance and surrender, operationalized in the Acceptance and Action Questionnaire (65); emotional breakthrough, operationalized in the Emotional Breakthrough Inventory (13); boundlessness, operationalized in the Altered States of Consciousness Questionnaire (17); and adaptive responses to challenging experiences, as operationalized in the Responses to Challenging Psychedelic Experiences Inventory (7) — all consistently predict therapeutic benefit, while their corresponding negatives (avoidance, suppression, resistance) predict diminished or adverse outcomes. We propose that these psychological constructs share a somatic substrate: the participant’s mode of interoceptive engagement with intense ascending, bottom-up signals from the body. One possibility is that acceptance has a partially somatic substrate when the body remains expanded under pressure, and resistance has a partially somatic signature when the body contracts. The clinical literature has named the consequence; the somatic substrate has been unspecified.

Finally, we consider ‘interoception as perception’ and ‘interoceptive engagement’ as capacities that can be trained. Later in this paper we define training in ‘receptive practices’ targeting ‘interoception as perception’, and also ‘generative practices’ targeting ‘interoceptive engagement’.

## 7 A theoretical model of how interoception training could improve psilocybin therapy outcomes

We propose a theoretical model in which two trainable capacities work together to produce a richer repertoire of bottom-up input available to correct top-down prediction errors, and thus improve clinical outcomes. The first capacity, interoception as perception, refers to the participant’s perceptual access to bottom-up bodily signals. The second, interoceptive engagement, enables the body to remain in an expanded stance toward those signals.

Ideally, during the dosing session, psilocybin relaxes top-down predictions while training allows for rich bottom-up input that meets a body in an expanded state. The expanded body — with regulated breath, autonomic flexibility, an expanded motor repertoire, and affective flexibility — is more able to remain organized in the presence of intense signals (e.g., emotions from past difficult experiences, ongoing threats, existential threat). An expanded bodily state may create conditions in which new affective and experiential responses become more available that contribute to adaptive coping and reduction of anxiety and depression symptoms.

Conversely, when the same pharmacological action of psilocybin meets a body in a contracted state — short breath, autonomic rigidity, frozen posture, affective distancing — the result is different. The participant may experience difficulty maintaining bodily and affective organization in the face of an onslaught of bottom-up signals, leading to feelings of overwhelm, defensive return to old maladaptive coping, and symptoms left unchanged or worse.

## 8 Mapping interoceptive modes to preparation training practices

The exercise choices we have made for our pilot study can be mapped onto a grid that makes the options explicit. Note that mapping the possibilities generates testable hypotheses about which signal type-by-engagement mode combinations are under-developed in psychedelic preparation more broadly. **Table 2** is constructed with a single practice in each cell as an illustration (rather than a comprehensive catalog of practices).

**Table 2:** Mapping practices to interoceptive modes.

*Signal type*	Engagement mode	
	Receptive	Generative
*Exteroceptive*	Mindful touch: contact on skin surface	Self-applied pressure with a hand
*Deep somatic*	Attention to muscular tension, soft tissue, MABT Stage 1	Yiquan standing meditation with isometric opposing forces
*Visceral*	Tuning into gut sensations, heartbeat, movements of diaphragm with in-breath	Pranayama breath manipulation
*Vestibular*	Lying down with attention to felt sense of orientation in space	Slow movement under load, as in tai chi
*Nociceptive*	Passive attention to pain, as taught in Mindfulness-Based Stress Reduction	Iyengar-style yoga postures held at edge of discomfort
*Emergent*	Body scan with whole-body breath sweep from toes and out of top of head	Qigong moving meditation with arm movement without load

## 9 Operationalizing the framework: an initial empirical test

The framework generates a manualized preparation protocol now being tested in BACK007, an IRB-approved Phase 2 trial of group retreat psilocybin therapy for patients with metastatic cancer that is currently enrolling. The trial adds a three-session arc of receptive and generative interoception training — two sessions delivered virtually and one in person at the retreat before dosing — to the Group Retreat Psilocybin Therapy model we have described (1). (Complete session protocols are provided in **Supplementary Material S1**.)

### 9.1 The three-session arc

Each session pairs a receptive component, targeting interoception as perception, with a generative component, targeting interoceptive engagement, and the sessions progress through the taxonomy from its most accessible category toward its most challenging. In Session 1, participants learn ‘feeling into the body’ through a guided body scan drawn from Mindful Awareness in Body-oriented Therapy in which the participant’s own hand provides a felt somatic focus at surface locations — beginning in the exteroceptive category because skin-level sensation is familiar and physical contact sends real bottom-up afferent signals that the participant is training themselves to detect (41). This process is distinct from the body scan taught in Mindfulness-Based Stress Reduction (66). The generative component is a standing meditation under gentle isometric load, produced by opposing forces through the body (the skeleton settling downward while muscles and connective tissue lift), held for two to three minutes with guided noticing of body and mind; its overarching question is: am I expanding or contracting under this pressure?

Session 2 extends the scan into deep somatic, visceral, and vestibular territory and closes with the participant moving attention back and forth between a tight area and an area of ease — the first stage of afferent anchoring, described below. The generative practice adds a slow, gathering arm movement with long nasal breathing to the standing hold, cultivating an open-chest, expanded posture as the participant’s default for the supine dosing position. In Session 3, delivered in person, the scan’s final phase turns to the nociceptive category — how the body has been affected by cancer and its treatment — and participants are offered a small buckwheat pillow as a physical anchor for a place in the body where ease can be found; the same pillow is available during the dosing session. The generative practice repeats with gentle, consented facilitator adjustment. Throughout the arc, descriptor flash cards drawn from the taxonomy’s vocabulary are introduced only after each experiential exercise, on the pedagogical principle that language deepens perception when it follows experience and can flatten it when it arrives first (**Supplementary Material S2**).

### 9.2 Afferent anchoring

When a participant encounters overwhelming feeling during the psilocybin experience, we coach them to move attention to a region of the body that feels neutral or at ease — or, if they can, into the part of the body involved — a skill we call afferent anchoring, introduced in stages across Sessions 2 and 3. We hypothesize that afferent anchoring functions by increasing attentional weighting on afferent signals (27, 28), providing alternative bodily input that can constrain escalating threat predictions from visceromotor cortices at a moment when psilocybin has already relaxed precision on high-level priors; the participant remains embodied — this is not dissociation or distraction — but the afferent channel feeding the brain’s ongoing interoceptive construction is different (Section 10; **Supplementary Material S4**). In one session, a participant on the verge of intense grief placed her hand on her chest and then asked a facilitator to place their hand over hers; coached to tune in to the warmth and gentle pressure, and then to feel deeper into the area of her heart, she was able to stay present and move into and through the grief in manageable steps rather than backing away from what had seemed too overwhelming to approach.

### 9.3 Provenance of the practices

The receptive practices are adapted from Stage 1 of Mindful Awareness in Body-oriented Therapy (MABT) (41), an evidence-based, manualized approach whose trials demonstrate that interoceptive awareness is trainable, with training-related plasticity in interoceptive neural networks (67) and self-reported improvements that correlate with mental health outcomes (68–70); we have drawn on MABT’s training sequence, qualities of touch, and home practice. The generative practices are adapted from yiquan, a Chinese internal martial-art lineage developed by Wang Xiangzhai, in which meditation is performed under sustained isometric load (71); the adaptation — including a seated alternative, holds foreshortened to two to three minutes, and explicit framing as preparation for receiving intense feeling rather than as martial training — was made by an author trained in this lineage (AM). Extended adaptation notes appear in **Supplementary Material S3**.

### 9.4 Pre-specified hypotheses

Three hypotheses were pre-specified in the trial design. H1: preparation that includes interoception training will measurably increase interoceptive capacity, assessed by the MAIA subscales of noticing, not-distracting, self-regulation, body listening, and trusting, measured pre- and post-training (47, 48). H2: increased interoceptive capacity will increase somatic granularity in post-session participant accounts, assessed by analysis of session and integration transcripts for references to the body and for use of granular somatic descriptors, with historical comparison to transcripts from prior group retreat cohorts who did not receive interoception training. H3: participants with greater post-training interoceptive capacity will report deeper dissolution experiences, measured by the MEQ-30, without an increase in challenging experiences, measured by the Challenging Experience Questionnaire.

### 9.5 Feasibility and acceptability

Alongside these hypotheses, we will measure feasibility (completion rates for each preparation session, time required for each exercise, and facilitator-reported challenges in delivery) and acceptability (participant-reported ease, usefulness, and comfort with each exercise, and open-ended responses about what participants found helpful and what they would change).

### 9.6 Limitations of this initial test

Several limitations of this initial testing are worth naming. The design is single-arm, with comparison to historical cohorts rather than a concurrent randomized control. The sample size supports feasibility and hypothesis-generating analyses rather than confirmatory tests of efficacy. The population — metastatic cancer patients in a group retreat setting — limits direct generalizability, though as argued in Section 2 this population may reveal a phenomenon present more broadly. Finally, facilitator training for delivering interoception training has not yet been standardized across settings, which will need to be addressed in further research.

## 10 Theoretical mechanisms: why interoception matters for psilocybin therapy

Our account builds on four empirically grounded theories of brain function that relate to the mechanism of psilocybin.

### 10.1 Relaxed beliefs under psychedelics in brief

Carhart-Harris’s REBUS (Relaxed Beliefs Under Psychedelics) posits that psilocybin relaxes the precision weighting the brain places on high-level prior predictions, allowing bottom-up signals to correct prediction errors (64). Notably, the term ‘prediction’ in this context refers to assessments generated by the brain that are fast, unconscious, and unintentional. (These ‘predictions’ are totally unlike the common use of the term to denote an intentional, deliberative speculation.) When a patient starts with high-level priors that are rigid (e.g., “I must keep working to be valued”), skewed (e.g., “dying is guaranteed to be too painful to bear”), or distorted (e.g., “my spouse won’t be able to bear this”), a psilocybin experience can allow those high-level predictions to be ‘relaxed’, which allows bottom-up signals from the body to update those predictions—in this model’s language, to correct prediction errors. What this looks like in clinical practice is that a participant goes into a psilocybin session holding rigid beliefs, and comes out realizing that they’ve changed—their beliefs have been revised—and they say things like “I am worthy whether or not I’m working,” or “dying might be hard but I have people and resources who can help,” or “maybe my spouse can share my sadness with me.” What REBUS does not directly address is the nature of bottom-up input and how that input revises the brain’s interoceptive prediction model.

### 10.2 Canalization and the post-psychedelic plasticity window

Carhart-Harris, Chandaria and colleagues recently proposed a complementary account that extends REBUS in a direction relevant to our framework. They propose that psychopathology can be understood as pathological canalization (72): repeated experience results in attractor states that are entrenched, self-reinforcing configurations of belief, affect, and behavior that resist updating. In their account, psychedelics act in part by transiently flattening this attractor landscape for a period of enhanced plasticity during and after the acute psychedelic experience. The contraction patterns we describe in Section 2 can be readily understood as canalized somatic configurations: bracing, guarding, and defensive reinterpretation of bodily signal that are reinforced over years of bodily threat, treatment, and patienthood. In this situation, somatic preparation practices taught before dosing, such as awareness of the inner body and the afferent anchoring skill, are then available during the period of enhanced plasticity that psilocybin initiates. Extending these practices into the integration period could prevent post-psilocybin reversion to old canalization patterns.

### 10.3 Seth’s interoceptive inference model

Seth’s interoceptive inference model posits that the self is an interoceptive prediction (27, 73, 74). The brain’s ongoing prediction of its own internal states is what constitutes one’s sense of self. For a cancer patient, their experiences of being diagnosed, their body surgically altered, their sense of well-being diminished by chemotherapy, would all change the stream of ongoing, involuntary predictions in a way that leaves that patient feeling that “I don’t feel like myself.” A psilocybin experience can disrupt these ongoing predictions. But this disruption cannot occur without a pre-psilocybin sense of what it feels like to be in this particular body—that interoceptive foundation is needed as a prerequisite for the disruption of selfhood. Seth’s model establishes why interoceptive capacity matters for the self-dissolution experiences that psilocybin can produce, but it does not specify the neuroanatomy through which interoceptive predictions are transmitted—a gap addressed by the model described next.

### 10.4 Barrett’s embodied predictive interoception coding model

A fourth mechanistic model, complementary to Seth’s interoceptive inference, specifies the anatomy through which interoceptive prediction is implemented. Barrett and Simmons’ Embodied Predictive Interoception Coding (EPIC) model (28, 75, 76) proposes that agranular visceromotor cortices send ‘top-down’ predictions to the body for the purpose of maintaining allostasis, the process the body uses to respond to stressors and maintain homeostasis. At the same time, these same predictions are transmitted to mid-to-posterior insula where they can enter conscious awareness as interoceptive sensations. These ‘top-down’ predictions may be modified by ‘bottom-up’ signals that arrive at the primary interoceptive cortex—where these bottom-up signals are compared against the ‘top-down’ predictions, and this process can result in ‘corrections’ of the ‘top-down’ predictions, called prediction errors, that are transmitted back to the visceromotor regions so that subsequent predictions can be updated.

Thus, what a person feels as interoception as perception is not just a raw, ‘bottom-up’, afferent signal—that bodily sensation is a combination of the top-down estimate about how the body is predicted to feel and a bottom-up physiological signal that may reinforce or correct or constrain the top-down prediction. Recent research using fMRI has confirmed that this complicated process is actually what happens in the human brain at the network level (77). EPIC ties interoception explicitly to allostasis (30)—the anticipatory regulation of bodily resources—and has been extended as a framework for understanding conditions of sustained allostatic–interoceptive overload in depression and across neurological and psychiatric illness (30).

The EPIC theory has two implications for the significance of interoception as a factor in psychedelic therapy. First, EPIC clarifies what it means for interoception to be impaired. The fidelity of interoception depends on the presence of bottom-up, afferent signals and also on how the brain handles prediction errors when these bottom-up signals are compared to top-down predictions. In situations, like illness, when the bottom-up signals are attenuated—whether by peripheral damage, chronic inflammation, or sustained allostatic load—top-down predictions can recirculate within the visceromotor-to-insula loop without being corrected by afferent signals from the body, causing mental and physiologic symptoms. Second, the EPIC theory clarifies that cancer-specific changes in the body’s capacity to transmit bottom-up signals create a vicious cycle that reshapes top-down predictions over time. The result is an interoceptive-allostatic system that issues top-down prediction based on defective body maps shaped by illness and treatment rather than from current bottom-up evidence.

## 11 Discussion

In this paper we propose a framework for somatic preparation in psilocybin therapy that distinguishes interoception as perception from interoceptive engagement, with expansion and contraction as two clinically central modes within engagement. We argue that cancer patients arrive for psilocybin therapy with both perceptual and engagement capacities altered by illness and treatment, that the empirical literature on psychedelic outcomes implicates engagement-related constructs as predictors of therapeutic benefit, but that current preparation protocols do not systematically address either capacity. In response, we offer a six-category taxonomy of somatic experience as a clinical scaffold for understanding what arises under psilocybin, map receptive and generative practices onto this taxonomy, and describe a pilot clinical trial as a first attempt to train both interoception as perception and interoceptive engagement. The framework is grounded in current predictive-coding accounts of interoception (REBUS, EPIC) but does not depend on any single neuroscientific model. Read this way, the framework offers an innovative therapeutic approach to the interplay between physical and mental health: a route by which patients estranged from their bodies by serious illness can be prepared to re-inhabit them.

Our interoception framework may also be read as a contribution to psychosomatic theory more broadly. Anxiety and depression associated with serious illnesses such as cancer are often conceptualized primarily as cognitive-affective syndromes, expressed through worry, sadness, demoralization, or altered meaning-making. While these dimensions are clinically important, we hypothesize that psychological distress in this setting may reflect not only maladaptive beliefs or emotional responses, but also altered bodily threat-monitoring, disrupted interoceptive signaling, and habitual modes of contraction or disengagement in how the patient meets internal sensation. From this perspective, psychological distress in cancer may also reflect disturbances in embodied prediction and related interoceptive processes. In this reading, anxiety and depression in cancer are not solely disorders of thought and affect, but may also involve disturbances of the embodied self as it predicts, monitors, and responds to bodily threat. This extends psychosomatic medicine’s traditional concern with mind-body reciprocity by identifying interoceptive engagement as a specific, potentially trainable therapeutic target.

What this framework means, if supported, for facilitators is that preparation may need to include efforts to strengthen a patient’s capacity for interoception as perception and interoceptive engagement. This approach asks facilitators to reframe the saying ‘psilocybin gives you what you need’ to ‘my responsibility is to prepare you and your body, offer guidance during the dosing session, and provide integration, because your response to a difficult experience may be an important determinant of the therapeutic outcome’.

Our principal contribution in offering this framework is conceptual. Most existing interoception research, dominated by heartbeat detection paradigms and broad self-report instruments, has not specified the dimensions of bodily experience that matter for psychedelic therapy. Existing clinical approaches to the somatic dimension, including Mindful Awareness in Body-Oriented Therapy (41), Somatic Experiencing (49), and Sensorimotor Psychotherapy (78), have developed sophisticated practices for cultivating interoceptive awareness but have not been purpose-built for the specific demands of psychedelic preparation, nor explicitly grounded in the predictive-coding mechanism that increasingly explains psychedelic action. Our framework draws on both lineages while making conceptual distinctions — the perception/engagement distinction, the receptive/generative practice categorization, the six-category taxonomy — that we believe are useful to further empirical research into ‘feeling into the body’.

Several limitations of our framework deserve naming. First, our distinction between interoception as perception and interoceptive engagement is based on clinical observation. The two capacities likely interact in ways our current account does not specify, and measurement work is needed to determine whether they are genuinely separable constructs or facets of a single integrated capacity. Second, expansion and contraction are presented as two clinically central modes within engagement, but the framework does not exclude additional modes (for example, equanimity, surrender, and other stances identified by contemplative traditions). Third, the taxonomy of somatic experience is a clinical scaffold rather than a psychometrically validated instrument, and its categories will require refinement as empirical work progresses. Fourth, the framework has been developed primarily for cancer patients in a group retreat setting, so its applicability to other populations and other delivery models remains to be tested. A final limitation is that our framework has been developed for, and its initial test is confined to, psilocybin; its extension to other compounds used or studied in patients with cancer is an open question. For example, ketamine’s dissociative effects may reduce, or alter, the relevance of interoceptive preparation, since dissociation attenuates bottom-up signals. Research with other psychedelics (such as MDMA or LSD) would be needed to test whether the framework articulated here for psilocybin can be generalized.

Our central hypothesis suggests a number of future directions for inquiry. The framework predicts that interoception as perception, in the types laid out by the taxonomy, and interoceptive engagement, should be measurable and modifiable. Measurement development in this area could be a fruitful collaboration between contemplative scientists, psychedelic practitioners and psychometric researchers. Our pilot study will provide initial evidence on whether the proposed training is feasible but further outcome testing will be needed. Our framework also has potential implications beyond psychedelic therapy. The contraction patterns we describe are not unique to cancer patients; they appear across chronic illness, trauma, depression, and aging. If our model is supported, interoceptive engagement may represent a transdiagnostic psychosomatic mechanism through which bodily threat-monitoring and embodied prediction shape psychological distress across multiple clinical conditions. This possibility suggests that somatic preparation approaches developed in psychedelic therapy may have broader relevance beyond the cancer setting. Finally, the framework raises questions about what facilitator training requires. For a facilitator to learn interoceptive engagement — particularly the generative mode under stress — is in itself an embodied skill that likely demands in-person, hands-on apprenticeship.

## 12 Conclusion

Psilocybin therapy for cancer patients is promising, but little research has been conducted on what makes preparation most effective. If psilocybin therapy produces therapeutic benefit through a dosing session in which patients experience a relaxing of their top-down predictions and more bottom-up interoceptive signals, then the body’s capacities for interoception are pivotal variables that influence outcome. We propose a framework that distinguishes interoception as perception from interoceptive engagement, identifies expansion and contraction as two clinically central modes of engagement, and offers a six-category taxonomy of somatic experience to organize both perception and practice. The receptive and generative training described here represents an initial attempt to translate this framework into a manualized preparation protocol. The BACK007 trial will provide first evidence on whether preparation that specifically addresses interoception is feasible and whether this preparation protocol produces any exploratory evidence that participant outcomes are improved. This pilot study is an early step in empirical inquiry into psychedelic preparation protocols that take the body seriously.

## Data Availability

The qualitative dataset that is quoted in this article is not publicly available. Requests to access these datasets should be directed to tonyback@uw.edu.
