# Supplementary material for: Preparing the body for psilocybin therapy: receptive and generative interoception in cancer patients
Source: Front Psychiatry. 2026 Oct 8;17:1890966. doi: 10.3389/fpsyt.2026.1890966 (PMC13687007; doi:10.3389/fpsyt.2026.1890966)
Supplement: Supplementary file 1 [file DataSheet1.pdf]

Supplementary Material

Preparing the body for psilocybin therapy: receptive and generative interoception in cancer patients

Anthony L. Back, Cynthia J. Price, Bonnie A. McGregor, Andrew Markell,

This Supplementary Material provides the operational detail of the interoception training protocol summarized in the main text. Section S1 gives the session-by-session preparation protocols; S2 describes the descriptor vocabulary training; S3 provides extended notes on the adaptation of practices from Mindful Awareness in Body-oriented Therapy (MABT) and from yiquan; S4 gives an extended description and clinical illustration of afferent anchoring. Category names and descriptor vocabulary refer to the six-category taxonomy presented in Table 1 of the main text.

S1. Preparation session protocols

The preparation arc comprises three sessions: two delivered virtually and one delivered in person at the retreat before the psilocybin dosing session. Each session pairs a receptive component, targeting interoception as perception, with a generative component, targeting interoceptive engagement. The sessions follow a deliberate progression keyed to the taxonomy, moving from the most familiar and accessible category (exteroceptive) toward more challenging territory (deep somatic, visceral, vestibular, and finally nociceptive), and from static holds toward movement and, in person, guided physical adjustment.

Session 1 (virtual)

Receptive component: guided sensation body scan with self-touch

Participants are introduced to 'feeling into the body' through a guided sensation body scan in which the participant uses their own hand to provide a felt somatic focus at a series of surface locations:

- base of the neck, each side
- top of each shoulder
- pectoral muscles
- solar plexus

This body scan differs from the body scan taught in Mindfulness-Based Stress Reduction, which typically asks participants to mentally attend to body regions without physical contact. Here the participant's hand actively engages touch and pressure receptors, sending real bottom-up afferent signals that the participant is training themselves to

detect. The exercise begins in the exteroceptive category because skin-level sensation is familiar and accessible — the 'feeling' modality closest to ordinary waking experience. Descriptive flash cards (Section S2) are introduced after the experiential exercise to build vocabulary for labeling what was felt.

Generative component: standing meditation under isometric load

The generative component is a standing meditation in which the participant learns to organize the body under stress. A gentle isometric load is produced by active engagement of opposing forces through the body: the skeleton settling downward while the muscles and connective tissue lift upward, creating isometric loading through opposing forces. The standing posture is held for two to three minutes. A seated alternative is offered to participants with musculoskeletal limitations (Section S3).

During the hold, participants are invited to notice what is happening in the body:

- What part of my body is working? Which muscles have come online?
- How is the breath responding?
- Where is there warmth, trembling, effort, ease?

And in the mind:

- What reactions, judgment, or commentary have shown up — catastrophizing, stories about being unfit, the urge to stop, judgments about doing it wrong?
- Notice the thought as a thought — not to argue with it, believe it, or push it away.

The overarching noticing question for the practice is: am I expanding or contracting under this pressure?

Session 2 (virtual)

Receptive component: extended body scan into deeper territory

The Session 2 scan extends 'feeling into the body' more deeply, adding somatic locations that cross into deep somatic, visceral, and vestibular territory:

- back of the head
- base of the neck
- chest and heart center
- solar plexus
- dan tien (lower belly)
- tailbone and sacrum

- hands
- feet

In the final phase of the exercise, participants are invited to locate one part of the body that feels tight or uncomfortable and another that feels more relaxed or at ease, and to move attention back and forth between these parts. This phase introduces afferent anchoring in its first, staged form (Section S4).

Generative component: standing meditation with slow gathering movement

The generative component repeats the standing isometric meditation of Session 1, now adding gentle, non-load-bearing movement of the arms. Our observation is that most participants arrive with a collapsed posture. Once the static hold has settled, the facilitator introduces a chest-opening movement: the arms open outward, expanding the front of the body, then draw inward toward the chest in a slow gathering motion. Instructional cues include:

- Move as if through water — slower than feels natural.
- Open the hands actively, engaging the tendons.
- Repeat the sequence several times.
- Throughout: long nasal inhale, long nasal exhale, so that the movement is also breath training — gradually expanding the capacity to use the breath while the body is under stress.

This sequence has a specific function for psilocybin preparation: during dosing, participants will be lying on their backs, and the open-chest, expanded posture is the default we want to cultivate for that position.

Session 3 (in person, at the retreat)

Receptive component: body scan extended to the nociceptive category

The Session 3 receptive component is a guided body scan similar to Session 2, with the final phase shifted to the nociceptive category. Participants are guided: “Bring your attention to how your body has been affected by cancer, by treatment, by everything you have been through.” They are guided to find one area where they can place a hand, notice what arises, and then move the hand to a place that feels more at ease.

Participants are offered a small buckwheat pillow to hold, so that the pillow can serve as a physical anchor for the place in the body where ease can be found. The same pillow is available to the participant during the psilocybin dosing session itself.

Generative component: standing meditation with facilitated adjustment

The generative component repeats the Session 2 sequence. Because participants and facilitators are together in person, gentle physical adjustment can be offered with the participant's permission — for example, the facilitator's hands resting on the participant's shoulders, allowing the participant to more easily feel upward expansion while the skeleton settles.

S2. Vocabulary training with descriptor flash cards

Throughout all three sessions, descriptive flash cards are used to build participants' vocabulary for labeling their felt experience. The cards provide perceptual categories drawn from the descriptor lists of the six-category taxonomy (Table 1, main text), so that participants can parse their experience with greater granularity than "good," "bad," or "intense" allows.

The pedagogical principle is that experiential exercises precede vocabulary introduction: we want participants to encounter a sensation first and then find language for it, not to approach the body through a pre-existing word list. Language deepens perception when it arrives after experience; it can flatten perception when it arrives first. The descriptor terms are offered as language that expands the range of 'in the body' experiences that participants and facilitators alike can identify, talk about, and make meaning from; they are not used to 'correct' participants or to enforce their 'proper' use.

The descriptor sets by taxonomy category are:

Taxonomy category	Descriptor vocabulary (flash card set)
Exteroceptive	tingling, prickling, buzzing, warm, cool, flushed, goosebumps, crawling, electric, itchy, tender
Deep somatic	heavy, dense, tight, clenched, braced, releasing, softening, melting, solid, trembling, unwinding
Visceral	churning, knotted, hollow, full, queasy, fluttering, dropping, rising, blooming, contracting
Vestibular	spinning, tilting, floating, falling, grounded, anchored, rooted, suspended, drifting, weightless
Nociceptive	sharp, burning, piercing, throbbing, crushing, tearing; bracing, guarding, flinching, freezing
Emergent	aliveness, stillness, spaciousness, luminosity, dissolving, oceanic, resonance, porosity, streaming

S3. Extended adaptation notes

S3.1 Adaptation from Mindful Awareness in Body-oriented Therapy (MABT)

The receptive practices are adapted from Mindful Awareness in Body-oriented Therapy (MABT) Stage 1, developed by Cynthia Price, and modified specifically for psilocybin preparation. MABT is a rigorously tested somatic awareness approach with a defined protocol for teaching interoceptive awareness, tested in populations often disconnected from their bodies due to trauma histories and substance use disorders. MABT research demonstrates that interoceptive awareness is a trainable skill, including evidence of training-related plasticity in interoceptive neural networks; across a range of clinical trials, MABT reliably improves interoceptive abilities measured by self-report, and these improvements correlate with mental health outcomes (references in main text).

Elements drawn from MABT for the present protocol:

- the foundational structure of the training arc
- practices of body literacy
- the use of guided attention to internal sensations
- the manualized sequence of practices
- the qualities of touch used in training
- the home practice component

Modifications for psilocybin preparation include the compression of the arc into three sessions keyed to the taxonomy, the use of the participant's own hand as the source of afferent signal in the virtual sessions, and the staged introduction of afferent anchoring in preparation for the dosing session.

S3.2 Adaptation from yiquan

The generative practices are adapted from yiquan, a Chinese internal martial-art lineage developed by Wang Xiangzhai in the early twentieth century, in which meditation is performed under sustained isometric load. The foundational principle of zhan zhuang standing meditation, as Wang Xiangzhai and his students have argued, is that structural engagement of opposing forces, combined with sustained attention, trains a capacity that purely receptive meditation does not train.

Elements drawn from yiquan for the present protocol:

- the standing posture, with the feet aligned along the outer edges of the shoulders
- the embracing arm position

- the principle of opposing structural forces — skeleton settling, connective tissue lifting — as the active mechanism of practice
- the slow gathering arm movement as a second teaching, introducing sensorimotor adjustment under continuous load

Adaptations for the clinical setting:

- a seated alternative for participants with musculoskeletal limitations
- explicit framing of the practice as preparation for receiving intense feelings in the body during dosing, rather than as martial training
- foreshortening of duration to two to three minutes per hold
- integration with the receptive sequence and with the afferent anchoring skill developed in parallel

This adaptation was carried out by one of the authors (AM), who has trained in this lineage.

S4. Afferent anchoring: extended description and clinical illustration

S4.1 The skill and its staged introduction

When a participant encounters overwhelming feeling during the psilocybin experience, we coach them to move attention to a region of the body that feels neutral or at ease — or, if they can, to move attention into the part of the body involved. We refer to this skill as afferent anchoring.

Afferent anchoring is introduced in stages across the preparation arc. It begins with the Session 2 receptive exercise that asks the participant to move attention back and forth between a tight area and an area of ease. It continues in the Session 3 receptive exercise, where the buckwheat pillow provides a gentle deep-tissue interoceptive stimulus and a physical anchor for the place of ease. The intent is that the participant arrives at the psilocybin session equipped with an embodied pathway for moving into informative afferent signal when descending predictions begin to spiral.

S4.2 Hypothesized mechanism

We hypothesize that afferent anchoring may function by increasing attentional weighting on afferent signals. This may provide the participant with alternative bodily signals that constrain escalating threat predictions from visceromotor cortices. Because psilocybin has already relaxed precision on high-level priors, the brain is unusually ready to update on the basis of this afferent input. The participant remains embodied throughout — this is not dissociation or distraction — but the afferent channel feeding the brain's ongoing

interoceptive construction is different (see Section 10 of the main text for the theoretical grounding).

S4.3 Clinical illustration

During one session, we worked with a participant on the verge of intense grief connected to leaving her partner. We had her place her hand on her chest, and a short time later she asked a facilitator to place their hand over hers. The facilitator coached her to ‘tune in’ to the warmth of her own hand on her chest, then to the gentle pressure of the facilitator’s hand, and then to feel more deeply into her chest and the area where she felt her heart. This allowed her to stay present with her grief, to experience it more fully with the felt support of the facilitator, and to move into and through the grief in manageable steps rather than backing away from something that had seemed, at the outset, too overwhelming to approach.
